# Development of a HPLC Method for Analysis of a Combination of Clofazimine, Isoniazid, Pyrazinamide, and Rifampicin Incorporated into a Dermal Self-Double-Emulsifying Drug Delivery System

**DOI:** 10.3390/mps6060104

**Published:** 2023-11-01

**Authors:** Daniélle van Staden, Richard K. Haynes, Frank Van der Kooy, Joe M. Viljoen

**Affiliations:** 1 Faculty of Health Sciences, Centre of Excellence for Pharmaceutical Sciences (PharmacenTM), Building G16, North-West University, 11 Hoffman Street, Potchefstroom 2520, South Africa; dvanstaden711@gmail.com (D.v.S.); or haynes@ust.hk (R.K.H.); frank.vanderkooy@nwu.ac.za (F.V.d.K.); 2 Rural Health Research Institute, Charles Sturt University, 346 Leeds Parade, Orange, NSW 2800, Australia

**Keywords:** Tuberculosis, clofazimine, high performance liquid chromatography, isoniazid, pyrazinamide, rifampicin

## Abstract

We describe the development and validation of a new high performance liquid chromatography (HPLC) method for analysis of a combination of the first-line anti-tubercular drugs isoniazid, pyrazinamide, and rifampicin together with clofazimine. This is a unique challenge since clofazimine and rifampicin are relatively highly lipophilic drugs, whereas isoniazid and pyrazinamide are considerably more hydrophilic. Thus, clear separation of peaks and quantification of four individual drugs can present difficulties during the development of an analytical method. Detection was established at two wavelengths—254 nm for isoniazid and pyrazinamide and 320 nm for clofazimine and rifampicin. Gradient elution was employed using 0.1% aqueous formic acid (A) and acetonitrile (B); clear separation of the four drugs was achieved within 10 min. A linear relationship was indicated by a correlation coefficient (r^2^) of 0.9999 for each anti-tubercular drug, respectively. The limit of detection (LOD) for the individual drugs was 0.70 µg/mL (isoniazid), 0.30 µg/mL (pyrazinamide), 0.20 µg/mL (rifampicin) and 0.20 µg/mL (clofazimine). Precision experiments rendered a mean recovery percentage of 101.25% (isoniazid), 98.70% (pyrazinamide), 99.68% (rifampicin) and 97.14% (clofazimine). This HPLC method was validated and is reliable, repeatable, and accurate for the purpose of conducting simultaneous HPLC analyses of the four anti-tubercular drugs.

## 1. Introduction

Tuberculosis (TB) is a significant threat to public health, as 20–40% of the global population is affected by this deadly disease [1,2]. Moreover, TB is still the leading cause of death caused by a single infectious disease despite the End TB Strategy of the World Health Organization (WHO) [3]. A potentially interesting approach that may aid the WHO strategy was recently reported that entails the introduction of clofazimine, which is mainly used for the treatment of leprosy, into first-line TB regimens in order to combat drug resistance, potentially shorten treatment times, and improve therapeutic outcomes [4]. Combining clofazimine with first-line TB drugs, in particular rifampicin, resulted in synergistic suppression and elimination of the growth of actively replicating bacteria [4]. Additionally, clofazimine exhibited improved efficacy when used in combination with isoniazid and rifampicin against slowly replicating mycobacteria, including those within biofilm-forming cultures [4]. These findings signify the potential advantage of adding clofazimine to first-line regimens due to its enhanced efficacy in eliminating slowly replicating bacteria [4]. Furthermore, synergism between clofazimine and pyrazinamide was demonstrated through oral administration of this drug combination, which substantially reduced the effective amount of clofazimine required, from 25 mg/kg to 6.25 mg/kg [5].

This synergism will be of particular advantage for developing a topical treatment for cutaneous TB as clofazimine is generally avoided due to dose-dependent skin discoloration [6,7]. This notorious dose-dependent skin discoloration is accompanied by other adverse effects such as severe gastrointestinal reactions (nausea, vomiting, abdominal pain, diarrhea), conjunctival deposition, and dark-brown pigmentation of fingernails. This is attributed to the accumulation of clofazimine in adipose cells in the heart, liver, breasts, adrenal glands, pancreas, spleen, bone marrow, and the lamina propria of the jejunum. The notably long half-life of clofazimine, ranging from 65 to 70 days, can be linked to the highly lipophilic nature of this drug. Overall, topical delivery of clofazimine will drastically decrease serious adverse effects, since systemic absorption is bypassed [8,9]. Furthermore, the use of decreased clofazimine concentrations will assist in the formulation of dosage regimens. Clofazimine has poor aqueous solubility and incorporating sufficient clofazimine into a solubilized dosage form is challenging [10,11].

**Figure 1:**
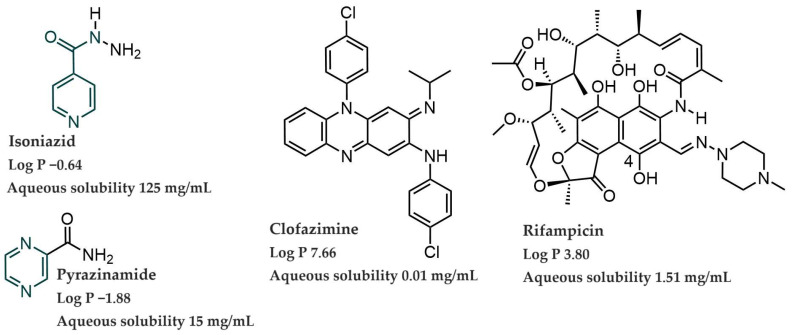
Structures of isoniazid, pyrazinamide, clofazimine, and rifampicin with their respective log *p* values and aqueous solubilities.

The structures of the four anti-tubercular drugs, isoniazid, pyrazinamide, clofazimine and rifampicin, discussed here are illustrated in Figure 1 together with their log *p* values and aqueous solubilities [1,8,11,12,13,14,15,16,17,18,19,20,21,22]. Moreover, rifampicin is amphoteric with a pKa of 1.7 due to the 4-hydroxyl group and a pKa_2_ of 7.9 due to the *N*-methyl piperazine nitrogen atom, with an isoelectric point at pH 4.8 in an aqueous solution [23].

High performance liquid chromatographic (HPLC) analysis of a combination of these four compounds with their large differences in polarity has not been reported. However, a HPLC method for the simultaneous analysis of isoniazid, pyrazinamide, rifampicin, and ethambutol has been published [24]. This technique employed gradient elution with a mobile phase comprising 20 mM monobasic sodium phosphate buffer with 0.2% triethylamine (pH 7.0) and acetonitrile, at a flow rate of 1.5 mL/min [24]. In this study though, we have to deal with the highly lipophilic clofazimine, which has a log *p* value of 7.66 that is substantially more lipophilic than ethambutol with a log *p* value of −0.14 [1,25].

Our objective was to develop and validate a rapid and reliable HPLC method to accurately quantify isoniazid, pyrazinamide, clofazimine, and rifampicin in combined formulations. This was required to evaluate the effects of different routes of administration and formulations, such as involving solid oral dosage forms and transdermal, or topical formulations. As is now described, method development and validation were conducted according to the International Conference on Harmonization (ICH) and Good Manufacturing Practice requirements and specifications [1,11]. Therefore, in the following sections, the materials utilized, the procedures followed to prepare the standard solutions, the gradient elusion applied for analysis, formulation of the spontaneous emulsions, and method validation are described and discussed. Certain conclusions could be drawn with regard to the accuracy, reliability, and repeatability of the method according to the ICH guidelines.

## 2. Materials and Methods

All chemicals were of analytical reagent grade. Deionized water, obtained from a Milli-Q system, was used for all experiments. Clofazimine, isoniazid, and rifampicin were gifts from Prof. Wilna Liebenberg, head of the Solid-state Pharmaceutical Innovation and Nanotechnology (SPIN) research group at the North-West University, Potchefstroom, South Africa. Pyrazinamide was purchased from Merck (Darmstadt, Germany). Acetonitrile and formic acid were purchased from Merck (Darmstadt, Germany) and Sigma-Aldrich (Johannesburg, South Africa), respectively. For HPLC analyses, a Nexera-i^®^ model:LC-2040C 3D Plus, a Phenomenex Luna^®^ 5 µm C18(2) 100A CC column 150 × 4.6 mm (Torrance, CA, USA), photodiode array detector, an injector module, and LabSolutions software (version 5.97 SP1) were used for analysis and data acquisition (Kyoto, Japan).

Stock standard solutions were individually prepared for each drug. Rifampicin and clofazimine standard solutions were prepared with acetonitrile, and standard solutions of isoniazid and pyrazinamide were prepared in water. All standard solutions were initially prepared as 1000 µg/mL solutions prior to being serially diluted with a mixture of deionized water:acetonitrile (1:1) to yield 7 drug concentrations. Gradient elution was carried out with mobile phase A comprising 0.1% aqueous formic acid; and mobile phase B consisting of acetonitrile. A flow rate of 1.0 mL/min was used with the initial mobile phase ratio at 7% B for 2.5 min, after which it was increased to 55% B at 6 min when a gradient shift of 0/100% [(A)/(B)] was maintained until 8 min. Hereafter, the gradient was readjusted to the initial gradient of 93/7% (A)/(B) until the end of the 10 min run time.

The generation of self-double-emulsifying drug delivery systems (SDEDDSs) involved the preparation of a primary emulsion comprising a mixture of Transcutol^®^ and polyethylene glycol (PEG) (9:1) as the internal oil phase, together with water and a surfactant phase containing Span^®^83 and Tween^®^60 (1:1). For clarity, the primary emulsion excipient ratios are expressed as 9:9:2, representing the internal oil phase:water:surfactant phase. After preparation of the primary emulsion, a predetermined quantity of the external oil phase (avocado oil) was added to the primary emulsion in small increments while stirring without heat application in order to achieve the formation of a homogeneous SDEDDS. The four drugs were included at a concentration of 2% of each individual drug, respectively. All samples were kept at an ambient temperature and all samples comprising rifampicin were stored in amber bottles, flasks, and vials to avoid photodegradation of the rifampicin. The SDEDDS was extracted by diluting 1 mL of SDEDDS up to 100 mL with methanol and was subsequently sonicated for 5 min. Samples were filtered with a 0.45 µm syringe filter into HPLC vials for analysis.

Validation of the method was performed by incorporating linearity, accuracy, precision, limit of detection (LOD), limit of quantification (LOQ), system stability, and repeatability. Linearity was calculated by constructing a regression plot of drug concentration vs. peak area response that enabled the generation of a regression equation for each anti-tubercular drug.

## 3. Results and Discussion

The individual regression equations are given in Table 1. Next, the correlation coefficient (r^2^) was calculated on the basis that a strong linear relationship is signified by a correlation coefficient of 1 [1,11]. Accuracy results are also displayed in Table 1. Accuracy parameters were in concordance through rendering recovery percentages between 98% and 102% (%RSD < 15%).

**Figure 2:**
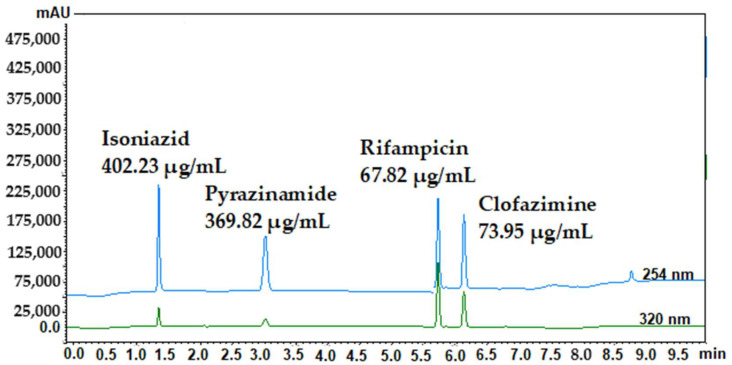
HPLC chromatographs of sample concentrations for isoniazid, pyrazinamide, rifampicin, and clofazimine, respectively. The top chromatogram was obtained using the photo-diode array detector at 254 nm (blue line) and the bottom chromatogram at 320 nm (green line).

The stability of the drugs was evaluated over a period of 24 h to ensure that no significant decomposition occurred during this time frame (Table 1). During the stability evaluation, no drug displayed concentrations that deviated more than 15% from the starting concentration. Therefore, all selected drugs were considered stable for a period of at least 24 h. System repeatability was verified by evaluating the peak area as well as the retention times of the drugs during six consecutive injections. All %RSD (percentage relative standard deviation) values were less than 2% (Table 1) and as a result, the method was found to be suitable for the analysis of these drugs. Figure 2 illustrates the HPLC chromatograms for isoniazid, pyrazinamide, rifampicin, and clofazimine, respectively.

It was decided to analyze rifampicin and clofazimine at a wavelength of 320 nm (Figure 1) as it allowed easier quantification when injecting decreased drug concentrations. The limit of quantification (LOQ) is the lowest concentration of drug that can be quantitatively ascertained, above which analysis is possible with the specified degree of accuracy and precision [1,11]. The LOQ is used particularly for determining impurities and/or degradation products [1,11]. The LOD, on the other hand, can be determined according to several methods, although the individual LOD concentrations were experimentally determined for the purpose of this study. The determined LOD was 0.70 μg/mL for isoniazid, 0.30 μg/mL for pyrazinamide, 0.20 μg/mL for rifampicin, and 0.20 μg/mL for clofazimine. Subsequently, the LOQ was determined as 2.31 μg/mL (isoniazid), 0.99 μg/mL (pyrazinamide), 0.66 μg/mL (rifampicin), and 0.66 μg/mL (clofazimine).

This method was applied to analyze the concentrations of the drugs incorporated into the SDEDDSs. Here, excipients that are frequently and safely utilized during formulations of dermal emulsions were considered, namely Transcutol^®^, PEG, avocado oil, water, Span^®^83, and Tween^®^60. The results obtained from the precision analyses of the SDEDDSs are given in Table 2.

**Figure 3:**
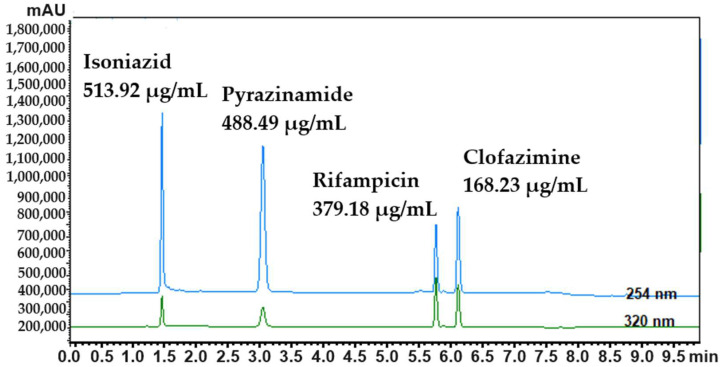
HPLC chromatographs of isoniazid, pyrazinamide, rifampicin, and clofazimine formulated into the transdermal/topical self-double emulsifying drug delivery systems. The top chromatogram was obtained using the photo-diode array detector at 254 nm and the bottom chromatogram at 320 nm.

As indicated in Table 2, the precision experiments involved measuring drug concentrations at three different concentration levels for each drug. The three different concentration levels for isoniazid were 250 µg/mL, 400 µg/mL, and 515 µg/mL; for pyrazinamide, 240 µg/mL, 370 µg/mL, and 490 µg/mL; for rifampicin, 180 µg/mL, 290 µg/mL, and 380 µg/mL; and for clofazimine, 90 µg/mL, 140 µg/mL, and 170 µg/mL. As the method complied with all predefined parameters, this overall method was established as precise and accurate. Figure 3 shows the HPLC chromatograms with clearly defined peaks for the four anti-tubercular drugs. These appear at the predetermined retention times (*cf.* Figure 2), with no interference from the different excipients used for preparation of the dermal SDEDDS formulations. In terms of inter-day precision, one concentration of each drug was analyzed each day for a three-day period. As the method complied with all predefined parameters, this overall method was established as precise and accurate.

As is evident from Table 2 and Figure 3, it was possible to achieve simultaneous quantification of isoniazid, pyrazinamide, rifampicin, and clofazimine. This method is therefore considered reliable and sensitive and complies with the ICH guidelines for method validation.

## 4. Conclusions

Validation parameters such as linearity, LOD and LOQ, accuracy, precision, sample stability, and system suitability were established. This method was also used to determine the sensitivity of the method when analyzing transdermal/topical SDEDDSs. The method was confirmed to be reliable for quantifying the four selected anti-tubercular drugs, with no interference from excipients generally used for the formulation of topical and/or transdermal products. Future work will include adding more anti-tubercular drugs that can be analyzed with a single method, since it is now established that multiple drug treatment regimens must be used for treatment of TB in order to suppress the emergence of drug resistance and maintain therapeutic efficacy. The proposed work will also be extended to include other excipients that are frequently utilized during the development of oral dosage forms of the individual drugs.

## Figures and Tables

**Table 1:** Validation parameters obtained for anti-tubercular drugs.

	Isoniazid	Pyrazinamide	Rifampicin	Clofazimine
Concentration range (µg/mL)	5.29–520.00	5.19–500.00	6.01–385.00	5.21–335.00
Regression equation	y = 924.51x + 259.47	y = 1154.40x − 1589.60	y = 4209.90x − 7194.10	y = 2431.60x − 624.93
r^2^ (>0.99) ^1^	0.9999	0.9999	0.9999	0.9999
Accuracy mean recovery (%) (%RSD) ^2^ (100 ± 2%)	100.04 (±0.06)	101.28 (±0.70)	100.15 (±0.80)	99.74 (±0.28)
Stability (%RSD) (<15%)	1.84	0.78	0.69	1.06
System repeatability (±SD) ^3^	1.46 (±0.18)	3.11 (±0.04)	5.71 (±0.16)	5.96 (±0.34)
Retention time (min) %RSD	0.34	0.94	0.88	0.23

^1^ r^2^ = Correlation coefficient; ^2^ %RSD = Percentage relative standard deviation; ^3^ SD = Standard deviation.

**Table 2:** Precision data obtained for anti-tubercular drugs (n = 9).

		Isoniazid	Pyrazinamide	Rifampicin	Clofazimine
Concentration range (µg/mL)		250–515	240–490	180–380	90–170
Repeatability (intra-day)	%RSD	0.03	0.02	0.01	0.03
Intermediate precision (inter-day)	Mean recovery (%)	100.30	100.95	99.91	98.91
	%RSD (day 2)	0.07	0.04	0.04	0.75
	Mean recovery percentage (day 2)	99.56	99.70	98.89	98.45
	%RSD (day 3)	0.02	0.01	0.17	0.05
	Mean recovery percentage (day 3)	101.25	98.70	99.68	97.14

## Data Availability

Data sharing is not applicable to this article.
